# Editorial: Structure and Function of F- and V-ATPases

**DOI:** 10.3389/fphys.2019.00358

**Published:** 2019-04-03

**Authors:** Paolo Bernardi, Giovanna Lippe

**Affiliations:** ^1^Department of Biomedical Sciences, University of Padova, Padua, Italy; ^2^Department of Food, Environmental and Animal Sciences, University of Udine, Udine, Italy

**Keywords:** F-ATP synthase, V-ATPase, mitochondria, lysosomes, metabolism

This Research Topic provides an updated view of rotary F- and V-ATPases, including aspects of their complex chemo-mechanical coupling and of their role in physiology and pathology. F- and V-ATPases are unique and ubiquitous rotary machines that evolved from a common ancestor and share the ability to pump H^+^ or Na^+^ powered by the energy deriving from ATP hydrolysis. The F-types (F-ATP synthases) also catalyze the synthesis of ATP using the electrochemical gradient of (usually) protons generated by respiration, providing most cellular ATP. Both F- and V-ATPases consist of a hydrophilic globular catalytic domain (F1 or V1, respectively) composed of an asymmetric hexameric ring with a central stalk located inside the ring; a hydrophobic membrane-embedded domain (Fo or Vo, respectively) which allows ion translocation across the membrane, and a peripheral domain with a stator function composed by one (in F-types) or three (in V-types) stalks.

In their fascinating review article, Singharoy et al. discuss a number of recently published crystal structures of the V1 sub-complex from *Enterococcus hirae* V-ATPase. This V-ATPase functions as a primary Na^+^ pump maintaining ion homeostasis and providing high salt tolerance to this organism The snapshots corresponding to different catalytic states of the rotary motor are then compared with the V1 pictures obtained by microsecond scale molecular dynamics simulations. Such comparison allowed to investigate the conformational transitions between catalytic intermediates and provided a unified model of the rotational mechanism. This shows similarities but also differences with that of the F-ATPases, such as the lack of sub-steps during each 120° rotation of the central shaft. Particularly relevant is the finding that, consistently with single-molecule experiments, the simulations revealed that the central shaft undergoes deformations, suggesting that it can store elastic energy.

The existence and the need for elasticity in rotary ATPases is further discussed by Colina-Tenorio et al., who present a detailed analysis of the latest high-resolution structures of the peripheral stalk of V- and F-ATPases, and also include A-ATPases, which in archaea function as the F-ATP synthases. These structures have defined the interactions of peripheral stalk subunits and their different conformations, confirming that the peripheral stalk is not static, as initially thought, but rather contributes (in part at least), to the overall enzyme rotational flexibility. An added bonus of this review is the thorough discussion of the role of the peripheral stalk in the process of dimerization of mitochondrial F-ATP synthase.

The relevance of conformational changes affecting the peripheral stalk in F-ATP synthase is also covered in the review of Starke et al. These authors discuss how catalysis can be inhibited by binding of the immunomodulatory drug Benzodiazepine (Bz)-423 to the OSCP subunit, which is located on the top of the peripheral stalk. Bz-423 specifically induced apoptosis of autoreactive B-lymphocytes in a murine model of *lupus erythematosus*, suggesting a promising approach for clinical treatment. The authors carefully describe their progress in defining binding of Bz-423 to OSCP through a wide variety of methods, including screening of a phage display library, genetic removal of OSCP subunit and NMR spectroscopy of purified OSCP and Förster resonance energy transfer in living cells, which allowed to show colocalization of the enzyme with fluorescently tagged Bz-423 with nanometer resolution.

The fundamental physiological function of F-ATP synthase in mammals is beautifully described by García-Aguilar and Cuezva. These authors summarize a large body of evidence demonstrating that the F-ATP synthase inhibitor IF1 is a main controller of oxidative phosphorylation under physiological conditions of oxygenation. Based on the findings described in this review, IF1 does only act as an F-ATP synthase inhibitor under conditions of oxygen deficiency and low pH, a view that is still widely held. Transgenic mouse models overexpressing IF1 in colon, liver or neurons displayed IF1-dependent F-ATP synthase inhibition capable of reprogramming energy metabolism to enhanced glycolysis and protection from the action of cytotoxic agents.

F-ATP synthase is vital to human health, and its malfunction has been associated to a variety of pathological conditions. These include mutations of mtDNA, which in humans encodes for Fo subunits a and A6L, which contribute to proton transport across the inner membrane. In their fascinating review, Dautant et al. summarize what is currently known about clinical syndromes induced by 58 mutations found in these mitochondrially-encoded genes. Most importantly, they also define the location of these mutations in the most recent high-resolution structures of F-ATP synthase, providing an eye opener on a new level of understanding of how individual mutations may affect enzyme assembly, structure, and catalytic mechanism.

V-ATPases are essential for numerous important processes in the eukaryotic cells where they mediate acidification of intra- and extra-cellular compartments. Their malfunction is related to several diseases, including cancer. In their very appealing review Collins and Forgac describe the peculiar modes of regulation of enzyme activity by *in vivo* assembly, i.e., by reversible dissociation of V1 and Vo domains in response to various nutrient cues, such as amino acid or glucose levels; and by enzyme targeting to different cellular membranes, which is controlled by isoforms of Vo subunit a. Most interestingly, these authors demonstrated that overexpression of the a3 isoform is responsible for plasma membrane targeting of V-ATPases in breast tumor cells, which leads to increased propensity to invade. The connections between V-ATPase and glycolysis in cancer is also covered in detail by Hayek et al., who focus in particular on how glucose signaling through V-ATPase acts as a molecular switch that dictates anabolic versus catabolic metabolism in mammalian systems. These glucose-sensing pathways appear to converge at the lysosomes, where a super-complex including aldolase, V-ATPase and the protein kinases AMPK and mTORC1 forms in response to the fructose 1,6 bisphosphate levels. This leads to inactivation of AMPK and activation of mTORC1, promoting cellular anabolism – a finding that can provide new targets against cancer.

We are very pleased with these reviews. We hope that our readers will enjoy them as much as we did, and that they will share our enthusiasm for one of the oldest and most important enzymes of life.

## Author Contributions

All authors listed have made a substantial, direct and intellectual contribution to the work, and approved it for publication.

### Conflict of Interest Statement

The authors declare that the research was conducted in the absence of any commercial or financial relationships that could be construed as a potential conflict of interest.

